# COntrolling NUTritional Status (CONUT) as Predictive Score of Hospital Length of Stay (LOS) and Mortality: A Prospective Cohort Study in an Internal Medicine and Gastroenterology Unit in Italy

**DOI:** 10.3390/nu15061472

**Published:** 2023-03-19

**Authors:** Emanuele Rinninella, Raffaele Borriello, Marco D’Angelo, Tiziano Galasso, Marco Cintoni, Pauline Raoul, Michele Impagnatiello, Brigida Eleonora Annicchiarico, Antonio Gasbarrini, Maria Cristina Mele

**Affiliations:** 1 Dipartimento di Medicina e Chirurgia Traslazionale, Università Cattolica Del Sacro Cuore, Largo F. Vito 1, 00168 Rome, Italy; emanuele.rinninella@unicatt.it (E.R.); antonio.gasbarrini@unicatt.it (A.G.); mariacristina.mele@unicatt.it (M.C.M.); 2 UOC di Nutrizione Clinica, Dipartimento di Scienze Mediche e Chirurgiche Endocrino-Metaboliche, Fondazione Policlinico Universitario Agostino Gemelli IRCCS, Largo A. Gemelli 8, 00168 Rome, Italy; pauline.raoul1@gmail.com; 3 Scuola di Specializzazione in Medicina Interna, Università Cattolica Del Sacro Cuore, Largo F. Vito 1, 00168 Rome, Italy; raffaeleborr@gmail.com (R.B.); dangelo.marco92@gmail.com (M.D.); tizianog.1993@gmail.com (T.G.); 4 UOC di Medicina Interna e Gastroenterologia, Dipartimento di Scienze Mediche e Chirurgiche Endocrino-Metaboliche, Fondazione Policlinico Universitario Agostino Gemelli IRCCS, Largo A. Gemelli 8, 00168 Rome, Italy; michele.impagnatiello@policlinicogemelli.it (M.I.); brigidaeleonora.annicchiarico@policlinicogemelli.it (B.E.A.)

**Keywords:** CONUT, disease-related malnutrition, length of stay, mortality, nutritional support, refeeding syndrome

## Abstract

Background: Hospital malnutrition affects nearly 30% of patients in medical wards and correlates with worse outcomes. An early assessment is necessary to stratify the risk of short-term outcomes and mortality. The predictive role of COntrolling NUTritional status (CONUT) score in this context has not yet been elucidated in Western countries. We aimed to test CONUT at admission as a predictive score of hospital outcomes, in an Internal Medicine and Gastroenterology Department of an Italian Tertiary Care University hospital. Methods: We prospectively enrolled patients admitted to our center, stratifying them into the four CONUT classes (normal = 0–1; mild = 2–4; moderate = 5–8; severe = 9–12 points) according to serum albumin (g/dL), total lymphocyte count (/mm^3^), and total cholesterol (mg/dL); the primary outcome measure was length of stay (LOS) and the secondary one was in-hospital mortality. Results: Out of a total of 203 patients enrolled, 44 (21.7%) patients had a normal status (0–1), 66 (32.5%) had a mild impairment (2–4), 68 (33.5%) had a moderate impairment (5–8), and 25 (12.3%) a severe impairment (9–12). The mean LOS was 8.24 ± 5.75 days; nine patients died. A moderate-severe CONUT correlated with a higher LOS at the univariate [HR 1.86 (95% CI 13.9–3.47); *p* < 0.0001] and multivariate analysis [HR 1.52 (95% CI 1.10–2.09); *p* = 0.01]. The CONUT score was also a predictor of mortality, with an AUC of 0.831 (95% CI 0.680–0.982) and with an optimal cut-off at 8.5 points. Nutritional supplementation within 48 h from admission correlated with lower mortality [OR 0.12 (95% CI 0.02–0.56) *p* = 0.006]. Conclusions: CONUT is a reliable and simple predictor of LOS and in-hospital mortality in medical wards.

## 1. Introduction

Hospital malnutrition represents an acknowledged risk factor for many adverse clinical outcomes [1,2], and the clinical management of malnourished patients is affected by higher in-hospital morbidity, mortality, and healthcare costs. A recent study demonstrated additional costs for hospital malnutrition of over $58 billion dollars in Western countries [3]. It is estimated that about 30% of hospitalized patients both in the United States and Europe present with malnutrition or risk of malnutrition at admission [1]. In Italy, a recent hospital report found over half of the patients at risk of malnutrition and over a third already malnourished at hospital admission [4]. Malnutrition is also an independent risk factor of poor postoperative outcomes in surgical patients [5] and has been linked to an increased risk of infections [6], significantly higher mortality for sepsis [7], a higher risk of pressure ulcers, and a worse outcome of wound healing [8,9]. In critically ill patients, major outcomes such as the duration of mechanical ventilation, the length of stay (LOS) in intensive care units (ICU), or infections are influenced by pre-existing malnutrition [10]. Hospital malnutrition maybe more evident in a Gastroenterology Department due to the role of the gastrointestinal tract in nutrients absorption [4]. However, despite these known associations, in daily clinical practice, hospital malnutrition remains often unrecognized, and the assessment of clinical nutrition of hospitalized patients is still underrated, probably due to a lack of awareness among clinicians, while focusing on diagnosis or treatment [11]. Several tools have been released by international societies for the screening—Nutrition Risk Screening 2002 (NRS-2002), Malnutrition Universal Screening Tool (MUST)—for the diagnosis of malnutrition, the most recent being the Global Leadership Initiative on Malnutrition (GLIM) criteria [12]. Despite a large diffusion among scientific sessions, the real application of such validated tools appears not sufficient in hospital settings, perhaps due to a lack of training, staff, and time [13]. The COntrolling NUTritional status (CONUT) score, a simple index calculated using serum routine analysis (albumin, total lymphocyte count, and total cholesterol) is associated with short- and long-term prognosis in several diseases [14]. The CONUT score has been proven not only to correlate with malnutrition grade [11] but also to have a high predictive value concerning clinical outcomes and morbidity. For example, in patients with cancer, a higher CONUT score predicts a lower overall survival, a lower progress/recurrence-free survival, and a lower cancer-specific survival after surgery [15,16], and a similar predictive value has also been observed for non-solid tumors and other hematologic disorders [17,18,19]. However, the CONUT score has also been investigated as a predictor of morbidity or mortality in various conditions other than malignancies, for example, in patients undergoing liver transplant [20] or heart bypass surgery [21], in patients with acute heart failure [22], or in patients with pulmonary embolism [23]. To date, fewer studies have been produced about in-hospital short-term outcomes such as the LOS or 30-day re-admission rates in medical units. A recent monocentric Chinese study performed by Hao in 2022 demonstrated that a higher CONUT score predicts a higher LOS and in-hospital mortality, specifically in patients with ischemic stroke [24]; another recent, large multicenter retrospective study performed in China in older adults, collecting data from more than eleven thousand patients, demonstrated that a higher CONUT score predicts a longer LOS and in-hospital mortality in elderly patients [25]. However, similar studies concerning LOS or in-hospital mortality in more heterogeneous cohorts of patients or in Western countries are still lacking.

Thus, we aimed to test CONUT at admission as a predictive score of hospital outcomes, such as LOS, in-hospital mortality, and 30-day re-admission rate in an Internal Medicine and Gastroenterology Department of an Italian Tertiary Care University hospital.

## 2. Materials and Methods

### 2.1. Study Design and Ethical Committee Approval

We performed a single-center, observational, prospective, cohort study. The study conformed to the Declaration of Helsinki and the norms of Good Clinical Practice. The Ethical Committee of Fondazione Policlinico A. Gemelli IRCCS, Catholic University of the Sacred Heart approved the protocol (code 2638/22). The STROBE guidelines for cohort studies have been followed [26].

### 2.2. Patients

Included patients were all adults (>18 years old) admitted to the Internal Medicine and Gastroenterology ward at the Fondazione Policlinico Agostino Gemelli IRCCS, Rome, Italy, from March 2021 to February 2022. All participants received information about the procedures to be performed in the study. Consent forms recording the agreement of patients to participate in the study were collected. Patients unable or refusing to give their consent to the study were excluded.

### 2.3. Protocol Description

Patients were assessed by the hospital staff (B.E.A. and M.I.) upon admission and then referred to internal medicine residents (R.B., M.D., and T.G.). Residents explained the protocol to the patients, requested informed consent, and collected data. Then, they collected demographic characteristics, primary diagnoses, and comorbidities; the registered date of hospital admission and discharge (or death, if any); clinical data; laboratory values; anthropometric—weight, height, and body mass index (BMI) —and other nutritional variables (i.e., NRS-2002, MUST, and nutritional supplementation). Due to the simultaneous presence of more diseases in this category of patients, the Charlson comorbidity index (CCI) [27] was calculated for each patient and preferred as a synthetic item instead of the single admission diagnoses. CONUT classes were defined based on serum albumin (g/dL), total lymphocyte count (count/mm^3^), and total cholesterol (mg/dL) as reported in Table 1.

The primary outcome measure for the present analysis was LOS and the secondary one was mortality during hospitalization. The re-admission rate within 30 days was also evaluated.

### 2.4. Data Collection and Statistical Analysis

Data were collected using a specific Excel© spreadsheet and shown using descriptive statistical methods. The Kolmogorov–Smirnov test was used to assess the normality of variables. Categorical variables were expressed as numbers (percentage) and continuous variables as mean ± standard deviation or median (interquartile range).

Patients were categorized according to total CONUT score into four classes (normal, mild, moderate, severe) and then grouped into two main classes (“normal-mild” and “moderate-severe”) for the inferential analyses. To estimate the risk of moderate-severe CONUT relative to normal-mild CONUT for the primary and secondary outcome measures, we used a multivariable logistic regression model. Kaplan–Meier curves were drawn, and the log-rank test was adopted to compare the obtained LOS intervals according to CONUT main classes.

A receiver operating curve (ROC) was constructed to provide the sensibility and specificity of CONUT to predict mortality. The optimal cut-off value of CONUT was calculated by applying the Youden Method to the constructed ROC.

A previous study reported an incidence of CONUT of more than 4 of 53.1% [28]. With a margin of error of 7% and a confidence interval (CI) of 95%, we estimated 196 patients to be enrolled to intercept the above-mentioned incidences (percentages).

We used the STATA^®^ Software (Version 14.0, Stata Corporation; College Station, TX, USA) to perform statistical analyses.

## 3. Results

### 3.1. Baseline Characteristics of Patients

Two hundred and three patients were evaluated, of which 127 (62.6%) were males and 76 (37.4%) females; the mean age was 66.05 ± 14.1 years. Most patients (68.5%) were admitted from the emergency department. The mean BMI (kg/m^2^) was 25.02 (SD ± 4.88) and the mean CCI was 3.02 (SD ± 2.43). According to NRS-2002, 70 patients (34.5%) were at risk of malnutrition. Conversely, according to MUST, 31 patients (15.3%) were at medium risk whereas almost half of the entire sample (48.7%) were at high risk of malnutrition. According to CONUT, 44 (21.7%) patients had a normal nutritional status (CONUT 0–1), 66 (32.5%) had a mild (CONUT 2–4), 68 (33.5%) had a moderate (CONUT 5–8), and 25 (12.3%) had a severe impairment of nutritional status (CONUT 9–12). The mean LOS in days was 8.24 ± 5.75; 38 (18.7%) patients developed a refeeding syndrome (RS); 9 patients (4.4%) died during hospitalization. All baseline data are shown in Table 2.

The CONUT classes (normal-mild vs. moderate-severe) correlated with age, admission type (elective or emergency), NRS-2002, MUST, the risk and occurrence of RS, the need for nutritional supplementation within 48 h from admission—either high-calorie and high-protein oral nutritional supplements (ONS) or artificial (enteral or parenteral) nutrition. As regards the main outcome measures, CONUT correlated with LOS and in-hospital mortality; re-admission within 30 days was not statistically different in the two groups (Table 3).

### 3.2. Associations of Risk Factors with LOS

**Figure 1:**
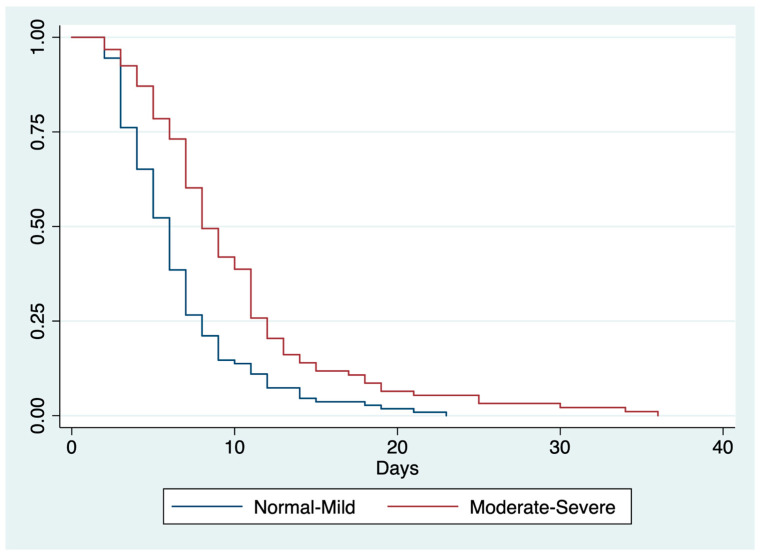
Kaplan–Meier curves for LOS according to CONUT score. Abbreviations: CONUT, controlling nutritional status; LOS, length of hospital stay; log-rank *p* < 0.0001.

Patients admitted with a CONUT score ≤ 4 had a lower mean LOS than those with a CONUT score ≥ 5 (6.5 ± 4.0 vs. 9.9 ± 6.4 days; *p* < 0.0001). At the univariate analysis, the ER admission, NRS-2002 > 3, MUST ≥ 2, a moderate/severe CONUT class, refeeding syndrome (RS) risk, and RS confirmed diagnosis were found to be risk factors for longer LOS. On the contrary, a normal-mild CONUT class was shown as a protective factor. In the multivariate analysis, ER admission, a moderate-severe CONUT score, and RS diagnosis were confirmed as independent risk factors of delayed LOS (Table 4). The Kaplan–Meier method confirmed different LOS curves between normal-mild and moderate-severe CONUT classes (*p* < 0.0001) as shown in Figure 1.

### 3.3. Associations of Risk Factors with Hospital Mortality

**Figure 2:**
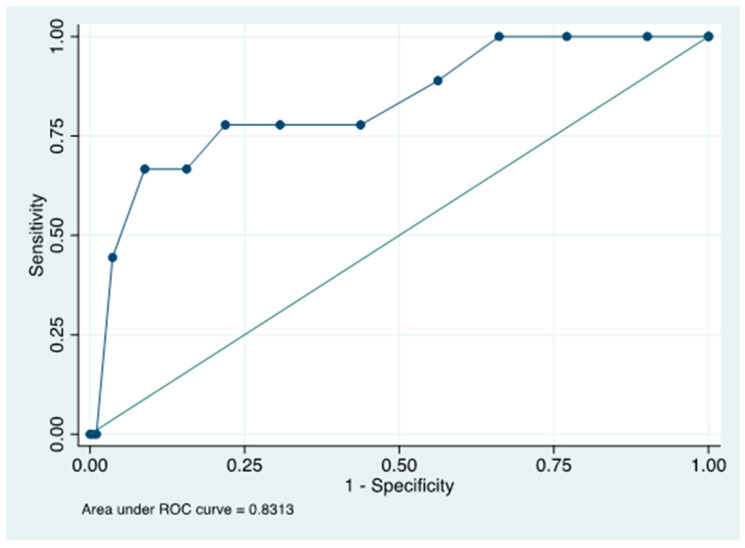
ROC curve for the prediction of mortality. Abbreviations: ROC, receiver operator characteristic.

Nine patients (4.4%) died during hospitalization. Higher CONUT scores and RS diagnosis were shown as potential risk factors for mortality in the univariate analysis. On the other side, a higher BMI was associated with lower mortality risk, as well as nutritional supplementation received within 48 h from admission (Table 5). Due to the limited number of death events in our study population (9), a multivariate analysis was not feasible. However, as reported at the ROC curve, the CONUT score was a reliable predictor of mortality, with an area under the ROC curve (AUC) of 0.831 (95% CI 0.680–0.982); the optimal cut-off obtained was 8.5 (Figure 2).

## 4. Discussion

After evaluating 203 patients admitted to an Internal Medicine and Gastroenterology Department, we demonstrated that the CONUT score can be a reliable predictor of higher LOS and in-hospital mortality. Indeed, at admission, patients reporting a CONUT score ≥ 5 points have nearly 90% probability of a longer LOS than those with a lower score. The predictive value of the CONUT score in assessing LOS was confirmed in the multivariate analysis. Interestingly, an NRS-2002 score > 3 (risk of malnutrition) and MUST ≥ 2 (high risk of malnutrition) showed an association with a higher LOS only in the univariate analysis. This is of interest, due to the objective nature of the CONUT score, based only on simple laboratory tests easily obtained in almost all clinical settings. Even if mortality events were only nine during hospitalization, univariate analysis confirmed a high CONUT score as a predictive risk factor of mortality, as also shown in the ROC curve. Thus, we can argue that a baseline CONUT value of 9 (or more) at admission predicts mortality during the hospital stay.

These results align with those of other reports investigating the role of CONUT in predicting LOS and mortality in several hospital settings, especially in elderly patients and in Eastern countries [22,24,25,29]. In details, Nishi et al. performed a retrospective analysis of a multicenter Japanese registry involving 838 patients (mean age 72 years) admitted for heart failure (HF): high CONUT scores were correlated with increased risk of in-hospital death in unadjusted and adjusted models and LOS [29]. Kato et al., analyzing data from a similar registry of patients admitted for acute decompensated heart failure (ADHF) (2466 patients, mean age 80 years), concluded that high CONUT scores were associated with higher in-hospital mortality and infection even when adjusting for other clinical covariates [22]. More recently, a Chinese study including patients admitted for acute ischemic stroke (AIS) (1079 patients, mean age 81 years) found a linear association between CONUT scores and LOS, and a significant association with hospital mortality [24]. Another retrospective study, analyzing data from 11,795 older adult Chinese patients found a higher LOS in higher CONUT classes and recognized CONUT (at the score ≥ 6) as the best predictor of in-hospital mortality among other five nutrition-related tools (including NRS-2002) [25]. Despite the lesser study population, we confirmed such evidence in a prospective cohort study, in Italy, in a different clinical setting (Internal Medicine and Gastroenterology department) and enrolling patients with a younger mean age (66 years). This confirms the reliability of the CONUT score as a predictive marker of short-term clinical outcomes irrespective of the geographical area and the population’s age. Indeed, the clinical value of CONUT resides in its simple laboratory data (albumin, cholesterol, lymphocytes count), reflecting the patients’ immunonutritional status. As regards albumin, it has been questioned as a proxy measure of nutritional status or total muscle mass, and rather indicated as a negative acute phase protein [30]. However, low albumin serum concentrations still have a predictive role in adverse outcomes in different clinical contexts of disease-related malnutrition, as demonstrated in recent studies [31,32]. Moreover, low serum albumin levels are associated with increased short- and long-term mortality in hospitalized patients, and serum albumin levels are an important predictor of in-hospital mortality or hospital complications in elderly patients [33]. On the other hand, the total lymphocyte count (≤1500 cells/mm^3^) may have a few limits due to other possible biasing conditions (i.e., hematological or infective diseases); however, recent studies on COVID have associated the total lymphocyte count with worse hospital outcomes and mortality in a context of severe inflammation [32,34]. Regarding total cholesterol, previous studies have associated low plasmatic levels with poor nutritional intake, systemic inflammation, and a worse prognosis in hospitalized patients, thus demonstrating a potential predictive value [35,36].

We did not find any difference between “normal-mild” and “moderate-severe” CONUT classes in terms of hospital re-admission within 30 days. This could be explained by the small number of re-admission events (8 vs. 5, respectively). Moreover, we do not register data about the re-admission type (if elective or by the emergency department), so we cannot make an inference about whether the re-admission could be related to malnutrition itself or other causes.

Our results highlighted the role of nutritional supplementation (received within 48 h from admission) in reducing mortality risk by nearly 90%. The nutritional supplementation included both high-calorie and high-protein ONS and artificial enteral or parenteral nutrition, according to the prescriptions of clinical nutrition team. This confirms the results of the EFFORT study, a multicentric randomized controlled trial, which demonstrated, in a large number of patients at nutritional risk, that an individualized nutritional support in medical inpatients could reduce adverse events and in-hospital mortality [37]. Moreover, in this study, we collected data about the occurrence of RS, since this work shared the same registry used for another of our study focusing on this topic, to which we remand for further details [38]. RS may occur when malnourished patients receive a prompt normocaloric artificial (enteral or parenteral) refeeding; it consists in a rapid shift in fluids and electrolytes in the intracellular space resulting in electrolytes abnormalities and cellular edema. It may have a dramatic impact in terms of morbidity and mortality, even if it is still underestimated and, as regards this study, it is significantly higher in the moderate-severe CONUT class. This confirms the efficacy of CONUT as a nutritional predictive score. 

The strengths of this study are homogeneous data collection and the prospective nature of the study. Moreover, to the best of our knowledge, this is the first Italian study on this topic. The main limitations are the monocentric design and the small number of deaths which does not allow us to perform a multivariable analysis, even if this demonstrated the efficiency of the department care. Thus, we think that CONUT value in predicting in-hospital mortality should be further confirmed in other similar prospective studies. Moreover, we did not perform a complete nutritional assessment since this study lacks data about body composition. Further studies are warranted to correlate the CONUT score with body composition parameters such as body cell mass or muscle mass. Finally, the impact of statin therapy (as regards total cholesterol) and the presence of hematological or infective diseases (as regards lymphocyte count) have not been investigated. 

Notwithstanding the above-mentioned limits, the study reflects the importance of using appropriate tools to stratify the nutritional risk at admission to the hospital, in order to prompt necessary nutritional interventions that could be effective in reducing mortality. Current guidelines [12] propose other nutritional tools such as NRS-2002, MUST, and GLIM Criteria, which are more standardized and focused on nutritional status. These tools investigate the amount and the speed of weight loss, the BMI, the reduced dietary intake, the severity of disease and, in the case of the GLIM criteria, also the loss of muscle mass. We also recognized the value of such an approach in clinical practice [4]. However, such a nutritional approach is still not widely spread until now in medical departments [12]. We thus decided to test another simple score as an objective and rapid method to predict prognosis. The CONUT score was demonstrated to be a simple, objective, and predictive method for this purpose, at least for hospital LOS and probably also for hospital mortality.

## 5. Conclusions

The CONUT score is a simple and reliable nutrition-related tool for stratifying the risk of higher LOS and predict mortality at admission. Given the relevance and ease of performing, health professionals should be incentivized to use the CONUT score in clinical practice to prompt personalized nutritional support. Indeed, we observed that early nutritional intervention (within 48 h of admission) could reduce in-hospital mortality.

The predictive role of different CONUT score cut-off values needs to be validated in populations with different diseases. Further studies are needed to confirm our preliminary results in large and multicentric medical cohorts.

## Figures and Tables

**Table 1:** Controlling nutritional status (CONUT) calculation.

Variables	Undernutrition Status			
	Normal	Mild	Moderate	Severe
Albumin (g/dL)	≥3.5	3.0–3.49	2.5–2.9	<2.5
Points	0	2	4	6
Total lymphocyte count (/mm^3^)	>1600	1200–1599	800–1199	<800
Points	0	1	2	3
Total cholesterol (mg/dL)	>180	140–180	100–139	<100
Points	0	1	2	3
Total CONUT score	0–1	2–4	5–8	9–12

Abbreviations: CONUT, controlling nutritional status.

**Table 2:** Patients’ baseline characteristics (n = 203).

Baseline Characteristics	Total (N = 203)
Males (n, %)	127 (62.6)
Females (n, %)	76 (37.4)
Age, years (mean ± SD)	66.05 ±14.08
Weight, kg (mean ± SD)	71.76 ± 16.29
Height, cm (mean ± SD)	169.03 ± 8.56
BMI, kg/m^2^ (mean ± SD)	25.02 ± 4.88
Admission type (n, %)	
Elective	62 (30.5)
Emergency	139 (68.5)
Other	2 (1)
CCI score (mean ± SD)	3.02 ± 2.43
NRS-2002 (n, %)	
>3	70 (34.5)
≤3	133 (65.5)
MUST (n, %)	
0	73 (36.0)
1	31 (15.3)
≥2	99 (48.7)
CONUT (n, %)	
Normal (0–1)	44 (21.7)
Mild (2–4)	66 (32.5)
Moderate (5–8)	68 (33.5)
Severe (9–12)	25 (12.3)
RS risk (n, %)	
Low	105 (51.7)
Medium	44 (21.7)
High	54 (26.6)
RS diagnosis	38 (18.7)
LOS, days (mean ± SD)	8.24 ± 5.75
In-hospital mortality (n, %)	9 (4.4)
Re-admission within 30 days (n, %)	13 (6.4)

Abbreviations: BMI, body mass index; CCI, Charlson comorbidity index; RS, refeeding syndrome; NRS, nutritional risk score; MUST, malnutrition universal screening tool; CONUT, controlling nutritional status; LOS, length of hospital stay; SD, standard deviation.

**Table 3:** Clinical and nutritional parameters according to CONUT classes.

Variables	CONUT	CONUT	*p*-Value
	Normal-Mild	Moderate-Severe	
	≤4 (n = 100)	≥5 (n = 103)	
Gender, male (n, %)	67 (60.9)	60 (64.5)	0.59
Age, years (mean ± SD)	63.9 ± 14.3	68.7 ± 13.5	0.01
Weight, kg (mean ± SD)	72.6 ± 16.1	70.8 ± 16.5	0.45
Height, cm (mean ± SD)	168.8 ± 7.5	169.3 ± 9.7	0.69
BMI, kg/m^2^ (mean ± SD)	25.3 ± 4.9	24.6 ± 4.8	0.27
Admission type (n, %)			
Elective	52 (47.7)	10 (10.9)	<0.0001
Emergency	57 (52.3)	82 (89.1)	<0.0001
CCI score (mean ± SD)	2.7 ± 2.4	3.3 ± 2.5	0.09
NRS-2002 (n, %)			
>3	21 (19.1)	49 (52.7)	<0.0001
≤3	89 (80.9)	44 (47.3)	<0.0001
MUST (n, %)			
0	54 (49.1)	19 (20.4)	<0.0001
1	17 (15.5)	14 (15.1)	0.93
≥2	39 (35.5)	60 (64.5)	<0.0001
Sodium (mmol/L)	140.9 ± 2.6	138.7 ± 4.6	0.0001
Potassium (mmol/L)	3.9 ± 0.4	3.8 ± 0.5	0.41
Calcium (mg/dL)	9.5 ± 0.6	8.8 ± 0.5	<0.0001
Chlorine (mmol/L)	104.0 ± 4.6	102.5 ± 4.5	0.04
Phosphorus (mg/dL)	3.3 ± 0.5	3.4 ± 0.6	0.64
Magnesium (mg/dL)	2.0 ± 0.3	2.1 ± 0.3	0.57
Albumin (g/L)	36.5 ± 4.4	26.9 ± 5.2	<0.0001
WBC (10^9^/µL)	7.4 ± 1.2	7.8 ± 0.8	0.63
Lymphocytes (10^9^/µL)	1.6 ± 0.4	0.9 ± 0.6	<0.0001
Total cholesterol (mg/dL)	166.6 ± 9.3	120.4 ± 10.1	<0.0001
Triglycerides (mg/dL)	106.2 ± 7.1	71 ± 6.8	0.001
Creatinine (mg/dL)	0.8 ± 0.1	0.9 ± 0.3	0.87
RS risk (n, %)			
Low	76 (69.1)	30 (32.3)	<0.0001
Medium	15 (13.6)	29 (31.2)	<0.0001
High	19 (17.3)	34 (36.5)	<0.0001
RS diagnosis			
Yes	11 (10.0)	27 (29.0)	<0.0001
No	99 (90.0)	66 (70.9)	<0.0001
Nutritional supplementation within 48 h (n, %)	27 (24.6)	47 (50.5)	<0.0001
LOS, days (mean ± SD)	6.5±4.0	9.9±6.4	<0.0001
In-hospital mortality (n, %)	2 (1.8)	7 (7.5)	0.049
Re-admission within 30 days (n, %)	8 (7.3)	5 (5.4)	0.58

Abbreviations: CONUT, controlling nutritional status; BMI, body mass index; CCI, Charlson comorbidity index; NRS, nutritional risk score; MUST, malnutrition universal screening tool; RS, refeeding syndrome; ONS, oral nutritional supplements; LOS, length of hospital stay; SD, standard deviation. Serum laboratory data are at admission. Calcium (mg/dL) is considered as the total serum calcium (calcium bound to albumin).

**Table 4:** Univariate and multivariate analyses of risk factors associated with LOS (n = 203).

	Univariate Analysis		Multivariate Analysis	
Risk Factors	HR (95% CI)	*p*-Value	HR (95% CI)	*p*-Value
Male	1.11 (0.83–1.48)	0.45		
Age	0.99 (0.98–1.01)	0.34		
ER admission	2.61 (1.89–3.61)	<0.0001	2.16 (1.48–3.16)	<0.0001
CCI score	1.01 (0.95–1.07)	0.65		
Baseline Weight	1.00 (0.99–1.01)	0.12		
Height	1.00 (0.98–1.02)	0.60		
Baseline BMI	1.02 (0.99–1.05)	0.08		
Baseline NRS-2002 > 3	1.47 (1.09–1.99)	0.01	0.90 (0.62–1.31)	0.61
Baseline MUST ≥ 2	1.57 (1.18–2.08)	<0.0001	1.05 (0.73–1.48)	0.81
Baseline CONUT				
Normal-Mild	0.53 (0.40–0.71)	<0.0001	Not included	
Moderate-Severe	1.86 (13.9–3.47)	<0.0001	1.52 (1.10–2.09)	0.01
RS risk	1.50 (1.13–1.99)	0.005	Not included	
RS diagnosis	2.21 (1.51–3.23)	<0.0001	2.00 (1.31–3.05)	0.001
Nutritional Supplementation within 48 h	0.74 (0.46–1.19)	0.21		
ONS	1.00 (0.74–1.37)	0.96		
Parenteral Nutrition	1.69 (0.96–2.97)	0.07		

Abbreviations: ER, emergency room; CCI, Charlson comorbidity index; RS, refeeding syndrome; BMI, body mass index; NRS, nutritional risk score; MUST, malnutrition universal screening tool; CONUT, controlling nutritional status; ONS, oral nutritional supplements. *p*-value < 0.05 means statistically significant.

**Table 5:** Univariate analyses of risk factors associated with in-hospital mortality (n = 9).

Risk Factors	OR (95% CI)	*p*-Value
Male	0.46 (0.09–2.29)	0.34
Age	1.04 (0.98–1.10)	0.14
ER admission	3.72 (0.46–30.44)	0.22
CCI score	1.12 (0.87–1.45)	0.34
Baseline weight	0.95 (0.89–1.01)	0.06
Baseline height	1.00 (0.92–1.08)	0.91
Baseline BMI	0.82 (0.69–0.97)	0.02
NRS-2002 > 3	2.48 (0.64–9.55)	0.18
MUST ≥ 2	0.37 (0.05–3.17)	0.37
CONUT	1.61 (1.21–2.15)	0.001
RS diagnosis	10.1 (2.4–42.6)	0.002
Nutritional Supplementation within 48 h	0.12 (0.02–0.56)	0.006
ONS	0.36 (0.03–2.17)	0.21
Parenteral Nutrition	4.75 (0.89–25.6)	0.07

Abbreviations: ER, emergency room; CCI, Charlson comorbidity index; RS, refeeding syndrome; BMI, body mass index; NRS, nutritional risk score; MUST, malnutrition universal screening tool; CONUT, controlling nutritional status; ONS, oral nutritional supplements. *p*-value < 0.05 means statistically significant.

## Data Availability

The data presented in this study are available on request from the corresponding author for any academic use upon citation of this article.
